# Health care in Bosnia and Herzegovina before, during, and after 1992–1995 war: a personal testimony

**DOI:** 10.1186/1752-1505-1-7

**Published:** 2007-05-29

**Authors:** Vladimir J Šimunović

**Affiliations:** 1 Mostar University School of Medicine, Mostar, Bosnia and Herzegovina

## Abstract

Market-based health care reform during democratic transition in Bosnia and Herzegovina was complicated by the 1992–1995 war, that devastated the country and greater part of its health care infrastructure. The course of the transition and consequences of war for the health system and health professionals are presented here from the perspective of the author. The description of real-life situations and their context is used to illustrate the problems physicians, as well as international community, were faced with and how they tried to cope with them during and after the war. Speaking openly about the mistakes that were made in those times is the first step in preventing them from happening again and an invitation for exchange of opinions and open academic discussion.

## Background

The year 1990 could be considered the beginning of what is known today as *the democratic transformation of political scene in the former Yugoslavia*. When the communist regime fell apart - together with the entire country after 50 years of rule - many people were attracted to aggressive nationalist and xenophobic political programs touted by most political parties in the newly-emerged countries. Due to lack of democratic experience, people were easily misled by nationalist symbolism and discourse of blame and soon, the stage was set for large-scale violence [1].

During this period, the international community seemed reserved and politely disinterested in the internal political developments in these countries, avoiding a determined or critical stance against even a possibility of having a war on European soil at the end of the 20^th ^century. Ultimately, the combination of international political inertia and national(istic) passions took its toll in human lives: the war broke out and hundreds of thousands of locals were killed, mutilated, crippled, tortured or raped, and millions still live in miserable conditions. During the 1992–1995 war in Bosnia and Herzegovina, a multiethnic country with Muslims, Serbs, and Croats as constituent nations, around 100,000 were killed, 300,000 wounded and mutilated, and two millions people became refugees [2].

The health care system in Bosnia and Herzegovina felt the dire consequences of the turbulent transition and war, from which it has still not recovered. What these consequences were and how the physicians, as well as international community, tried to cope with them during and after the war is presented here in the form of the first-hand account and through a description of real-life situations and their context. Hopefully, some conclusions may be drawn and lessons learned from the mistakes that were made in those times not so long ago, and maybe they can prove, if not practically useful, than at least interesting and stimulating for discussion.

## Health care system in Bosnia and Herzegovina before 1992–1995 war

### Organization

During communist rule, the health care system in the former Yugoslavia was centralized. Primary health care, provided by general practitioners at municipal health centers and their outpatient facilities, secondary health care was provided at both municipal health centers and regional hospitals, while tertiary level health care was provided at teaching hospitals linked to universities. Public health was organized through municipal, regional, and national institutes [3]. Health insurance was state-controlled and literally everybody had complete health protection. With the arrival of democracy, this structure of health care system remained more or less the same. The only thing that changed were the people occupying the key positions within the Ministry of Health and health care system and the criteria for their selection and appointment: ethnic and ideological affiliations were suddenly more important credentials than professional competence, knowledge, and experience. Unfortunately, this state of affairs has continued to the present time.

### Physicians

The standards and skills of the clinicians in former Yugoslavia were mostly satisfactory. Routine diagnostic and treatment procedures were performed in accordance with the standards applied in far more developed countries. Although there were not many internationally renowned physicians, not a few received at least part of their training in the best medical centers in the world. The main health indicators, such as newborn mortality rate of 14.5/1,000 live births, were comparable to those in West European countries [3]. Others, such as comatose patient treatment results, were even better than in many major medical centers in the world [4-9].

Most physicians belonged the middle or higher middle class as government salary was high enough to allow them to lead a comfortable life. Demanding additional financial reward from patients for medical service was illegal, immoral, and as a rule resulted in peer ostracism. To the best of my knowledge, corruption in hospitals in Sarajevo, capital of Bosnia and Herzegovina, and Central Bosnia was sporadic and insignificant. In short, medical doctors were a satisfied, socially respectable group with secure jobs and a comparatively privileged lifestyle.

## Health care system in Bosnia and Herzegovina during 1992–1995 war

### Reorganization attempts

Despite heated political rhetoric, nobody in the health care system really expected a war. No preparations were made, no stockpiling of medications, no reorganization plan to help us quickly adapt to wartime conditions – if the need arised. As a result, the hospitals in Sarajevo ran out of basic surgical material (dressings, bandages, sutures, cleaning solutions, and similar) within the first three months of the siege. Essential medications, oxygen, and anesthetic gases were at a premium, and the power and water supply were cut off after several months.

When the war started, the first organizational move was to replace all but a few hospital's and department heads with ethnically and politically suitable individuals of dubious professional and organizational abilities. The 'C*risis Headquarters,' *responsible for the organization of medical services in war conditions, were also dominated by aggressive and incompetent people, whose main qualifications were the ability to ardently express nationalistic, patriotic, and religious sentiments and a lack of any serious ethical restraints. For example, the post of Minister of Health was assumed by a semi-retired professor of dental medicine, specialized in prosthetics. In the winter 1992/93 when my patients were dying of cold and malnutrition as well as of wounds and my staff was not in much better condition, I paid him a visit to ask for assistance with organizational and logistic matters, as I was Deputy Surgeon General at the time. I found him sitting in his overcoat with the gloves on in a cold, empty office with broken windows, the only task on his empty desk that he had apparently been systematically tending to being an almost empty bottle of whiskey. He was drunk and I was obviously left of my own devices.

### Physicians

When the war began, the health professionals divided into two groups: the one that stayed and the other one that left. The estimates are that the number of people employed in the health care sector dropped from around 19,300 in 1991 to 11,857 in 1996 [10]. By the end of the war in the south-west part of the country, the number of local physicians and nurses had decreased by 1,200 and 3,752, respectively [10-12]. None of them were either prepared or trained to work under war conditions.

Becoming a wartime physician overnight was not an easy task. As medicals students, undergraduate as well as graduate, we were only taught how to be life-long learners, clinicians, educators, researchers, and managers [12-17]. We were not taught any skills that would be useful in war. However, we were about to enter a four-year military medicine course, and some of us found ourselves in the roles that we never expected to play.

### The accidental hero and negotiator

In war, the rules of civil society do not apply. Our unquestionable right to live suddenly becomes very questionable when we realize that authority and power lie in the hands of those with guns. And there were many of "*those with guns*" wandering around the hospital, enraged, drunk or drugged, barging in everywhere, even the operating rooms during surgeries. We were scared to death, but we at least had to pretend to have the situation in the hospital under control. Gaining respect from other players in that mad game was the only way to keep the work going. I hope the following vignettes will illustrate well the range of roles we had to play.

Just a few days before the war started, I was called to the hospital shortly before midnight to attend to two patients with gunshot wounds to the head. When I arrived to the Intensive Care Unit, the two men were in a coma, while a third one, a man with missile injuries to the arm and leg, lay in bed like dying royalty, surrounded by eight of his armed henchmen who obviously had little trust in physicians and wanted to supervise the surgery on their leader. I pretended to be angry and demanded that they leave the hospital. In response, they pointed their guns at me saying me that I would be the one to leave, for good. When I said that in that case they could perform the surgery themselves, their leader waved them off and I lead them all out like a group of badly behaved schoolboys, followed by the astonished looks from the hospital staff and a guard. After the surgery, around 4 a.m., when I decided to make the last round and then catch an hour of sleep, I was told that a hundred more like them surrounded the hospital and were ready to come in, and the only person they would talk and listen to was me.

Certainly, heroic ultimatums such as that one did not always work. Often, one had to resort to lengthy negotiations, for example, with a leader of a paramilitary unit (in the beginning, every block had its own paramilitary structure) who got it into his head to come to the hospital and take away a wounded patient for torture and execution. These negotiations, I am proud to say, were almost always successful, although they still continue in my nightmares.

## Health care system in Bosnia and Herzegovina in 1995–2005 period

The new political organization of Bosnia and Herzegovina, a political experiment devised by the best political minds in the world, has resulted in the degenerate structure whose only immediate effect was the expansion of bureaucracy. One half of the country, called the Federation of Bosnia and Herzegovina, has been divided in 10 cantons, each governed by an independent government. For the health care system this meant ten more ministers, each with his or her own entourage of deputies, aides, counselors, and technical staff. The other half of the country fared much better – they have only one minister. Recent statistics shows that 65% of an already paltry national income is spent on the administration. If we count in the lack of financial transparency, not much is left for other needs of the people of Bosnia and Herzegovina.

After the war, various international health organizations, governmental health agencies, and countless non-governmental organizations entered the scene. Both western and eastern oil-rich countries were earnestly declaring their intention to pour dollars into the devastated health care system and build a new one, better than any other in the world. Everybody was determined to implement nothing less than "*the world's best practice*" and "*European standards*."

### Physicians

It is difficult to believe how quickly the *heroes in white*, as journalists used to call us, transformed themselves into in an interest group offering minimal service for maximal gain under the new market rules, while showing little compassion for the impoverished population. A large number of private practices, some legal but most illegal, opened, charging the same fees to the haves and the have-nots, the first group representing only 5% of the population. Even professional solidarity among colleagues disappeared.

There are no statistics on how many health professionals left the country, but it is certain that among them were many of the very best. In their place came whatever human resources were left in the country. Again, the political and ethnic affiliation played a more important role then professional competence, especially when it came to key positions.

## International community: heroes, experts, money-makers, and saints

### Prewar period

Before the war, foreign experts rarely visited our country and our experience with international organizations was very limited as there was no need for humanitarian aid. The medical community for the most part had little interest in the activities and programs of organizations such as the World Health Organization and Red Cross. Likewise, as there were neither exotic infectious diseases nor epidemics in our country, and we had no extra money to invest in international health campaigns and *projects*, we received only scant attention from international organizations. This changed with the outbreak of war.

### War period

Almost with the first shot that was fired, various international organizations and representatives of international community started arriving on the scene. The show had begun. It is still going on, although on a much smaller scale than before as the attention has shifted to Afghanistan and Iraq. Very few pennies are sent these days to Bosnia and Herzegovina, but during the war *the locals *and *the internationals *had quite an intense relationship that went through several stages: from fascination and uncritical cooperation to mutual loathing and blaming. Perhaps describing them in more detail may prove enlightening to the reader.

#### Stage of fascination

The internationals started arriving as early as May of 1992. Telling them from the locals was not too difficult: they were fresh-faced, well-fed, dressed in something resembling a safari outfit, with helmets, bulletproof vests, and cameras. They were inquisitive, full of energy and elaborate questions, such as "*How do you feel when you treat children with brain wounds?" *or "*Are you scared?" *or "*Do you hate the people who are shooting at you?" *And they were clean – really clean. The only thing we truly envied them was their access to water. They could have a shower whenever they wanted to, while we barely had enough to drink. As in most wars, water is a most valuable asset.

Who were these internationals? In the beginning they were mostly politicians, journalists, and professional observers. It was only many months later that we began to understand the concept of secret services, clandestine work, and fox-hunting enthusiasts in search for new thrills. Thanks to my position in the main trauma centre and my eloquent pidgin English, I missed few of the visiting nobility and had the honor of shaking hands with French ministers, high-ranking United States civil servants, and English knights and royalty. It seems that the highlight of their visits was always the department where children with bullets in their heads were treated. The visitors would all be terribly upset by what they saw, express their deepest sympathy, promise us the moon, and then leave in a day or two never to return.

#### Stage of uncritical cooperation

The truth is that locals were thankful, though a bit confused and embarrassed, for the international and media attention they received. Between looking after the injured and taking care of the dead day in day out, they would suddenly see themselves on CNN, BBC or Sky News, explaining to concerned journalists how they had not had running water for weeks or shaking hands and exchanging smiles with this or that celebrity. To be fair, the visitors loved us, too. Why wouldn't they? They had never met so many tired, hungry, dirty people who could get killed any moment and who still smiled, behaved politely, and were so eager to please.

The real humanitarian workers who started bringing food to the city mainly worked for the UNHCR. Most of them were hired hands without much thought for what they were doing. They were not that interested in niceties either and did not really want to know how we felt under sniper fire. They would simply come with their trucks, unload whatever they had to unload, and leave as quickly as they could. Most of the time they brought useful items. Sometimes, however, they brought out of date food, antimalarial drugs, condoms, or dog and cat food. The days of *the internationals with projects *were still far away.

#### Stage of mutual loathing and blaming

High hopes and too many broken promises led to bitterness in the local population. Even the most benevolent and sympathetic internationals were perceived with contempt. At that time, most of the accusations thrown at the internationals were unfair: hundreds of small humanitarian groups were roaming through the country, often risking theirs lives to help local people. However, the results were poor – not because the humanitarians had bad intentions, but simply because they were unprepared either for the task or for the thieving instincts of the local population. Most of them were members of various religious and church groups, sweet and humble older people, who could barely find their way through a labyrinth of shelves in a supermarket, let alone the maze of Bosnian forests and all the fighting parties.

### Postwar 1995–2005 period

The Dayton agreement, signed in 1995, ended the most brutal part of the war. Everybody in Bosnia and Herzegovina was happy that the war was over; nobody was satisfied with the results. Nobody won, except for the criminals and war profiteers. The people definitely lost. They did not understand why the war was needed in the first place, much less who needed it. Apparently somebody did, as always. The talks about rebuilding and reconstruction of the country started immediately. It appeared that the international community finally opened its eyes, felt a pang of conscience and decided to do something to make up for all the destruction and pain it has allowed to last for four long years. Right.

The people in Bosnia and Herzegovina had, and some still have, two main misconceptions about the past events. The first one was that we were poor innocent victims. The second mistaken belief was that somebody, preferably everybody, was going to make it up to us.

### Reconstruction

The international community sent us experts in every field of human activity, from archaeological conservation to perfume production, to help us rebuild what was destroyed. The health sector was again at the top of the priority list. No more old crackers, condoms, and pet food. Billions would be invested in the reconstruction of the country, we just had to ask and the United Nations, European Commission, NATO, Stability Pact, OSCE, everybody would help the good and brave Bosnian people, innocent victims who suffered so much and were neglected by the world for so long.

The country was flooded with the internationals once again. But this time, they were not self-taught hippy-like humanitarian-aid workers or good-hearted church-going ladies. They were professionals from the upper echelons of society: retired professors mainly in public health, ex-Surgeon Generals, ex-presidents of this and that, Heads of Regional Offices, Principal Investigators, and so on. The total reform and reconstruction of health sector was under way or at the very least the reform of public health services, or primary health care, or health financing, or health insurance policy.

The international experts traveled only first class, slept only in the best hotels, ate the best food, drove in the most expensive cars. After all, they were risking their lives by visiting the country that had just been through a war. With daily fees ranging US$500–1,000, they could afford it. The fee for *local experts *was also high, increasing from US$20–50 a day during the wartime to as much as US$100 for exceptional work in the postwar period.

The magic word that opened all doors was "project." In those days, anyone with a modicum of self-respect had to have a project. Rushing to submit their project proposals, the experts would sometimes forget to change the name of the country in the project title and we would suddenly have to decide on a Breast Feeding Campaign in Moldavia or AIDS Prevention in Georgia. But, mistakes happen, no harm done. There were a lot of other projects, such as Doctors' Associated against the Torture and International Physicians against Nuclear War (although we did not have one), and Role of Nurse in the Sequence of the Rape, and of course – anti-smoking projects. If somebody wanted to help but had no idea what to do, a non-smoking campaign always came in handy. We had at least a hundred anti-smoking actions of all sorts. Millions of dollars were spent, but to no avail. The locals still smoke. It does not matter that at this moment only 30% of the inhabitants of Bosnia and Herzegovina have a safe water supply.

But the goose that laid golden eggs turned out to be medical education. In primary health care and family medicine alone we had at least ten programs and projects developed in USA, Canada, UK, Spain, France, or Greece to provide medical knowledge and training. On one occasion, a distinguished German professor of biochemistry submitted an education project proposal and listed three out of eight possible topics in which he proposed to retrain and update (another magic word) the local health professionals. The suggested topics were "*Heart*," "*Liver*", and "*Brain*". The choice of the remaining five topics on which we would like to be updated was ours. He also suggested that he would be prepared to train all of us – doctors, nurses, specialists, and students – together [18] for no more or no less than half a million US dollars. When I expressed my concerns and refused to support the project, he turned into a mortal enemy, one of the many I was later to make for mostly similar reasons.

## Concluding remarks

Today we witness the emergence of new academic disciplines, such as Peace through Health [19,20] where physicians are developing interventions by which they could prevent and heal the consequences of war. Such disciplines may be considered academic in the world that has not experienced a war, but in the country like Bosnia and Herzegovina, healing war wounds is a matter of daily clinical practice.

Our war experience has shown that intentions and actions of different profiles of international organizations and individuals trying to help the population and country affected by war have not been as effective as we, or they themselves, wanted and expected them to be. The specific characteristics and needs of the population have not been accurately identified and taken into account, and the greater part of the promised aid did not reach the affected at all. Therefore, the quality control of the proposed aid projects should be increased and they should be assessed for feasibility before put into action. Also, those for whom the aid is intended should be more actively involved in the realization of such projects as they probably know what kind of help they need and how they need it.

In the end, I hope that this testimony, although it is not evidence-based, may contribute to the development of all projects aimed at helping people and countries affected by war.

### Contributions

VJS wrote the draft of the paper and approved the final version of the manuscript. VJS is the guarantor of the study.

## Competing interests

The author(s) declare that they have no competing interests.

VJS worked as head of neural trauma service in Sarajevo war Hospital and in Mostar war hospital during 1992–95 war, as Deputy Surgeon General in Sarajevo (1992–93); as Deputy Minister of Health, Croatian Republic Herzeg-Bosnia (1993–94); as Adviser to Minister of Health of Federation of BH (1995–2000); as Team Manager in health related projects of World Bank in BH 1999–2000), as vice-dean for science at School of Medicine Mostar University (1997–2003) and as vice-dean for science at School of Health Study Mostar University (2004-present). He was the Principal Coordinator of European Union Tempus project for development BH medical libraries network (1998–2002) and he is the Principal Coordinator of European Union Tempus project "Dictum" for curriculum development in all BH Schools of Medicine (2003–2006) and Tempus project "Refine" for curriculum development in BH Schools of Nursing (2006-present).
